# Six‐ and Twelve‐Month Changes in Body Composition and 24‐h Energy Expenditure After a Very Low‐Calorie Ketogenic Diet

**DOI:** 10.1002/oby.70236

**Published:** 2026-06-15

**Authors:** Alessio Basolo, Paolo Piaggi, Valentina Angeli, Paola Fierabracci, Chiara Bologna, Giordano Paolucci, Guido Salvetti, Luca Chiovato, Jonathan Krakoff, Alberto Landi, Ferruccio Santini

**Affiliations:** ^1^ Obesity and Lipodystrophy Center, Endocrinology Unit 1 University Hospital of Pisa Pisa Italy; ^2^ Department of Information Engineering University of Pisa Pisa Italy; ^3^ Istituti Clinici Scientifici Maugeri IRCCS Pavia, PV Italy; ^4^ Obesity and Diabetes Clinical Research Section National Institute of Diabetes and Digestive and Kidney Diseases, NIH Phoenix Arizona USA

**Keywords:** energy expenditure, ketogenic diet, obesity, thyroid hormones

## Abstract

**Objective:**

This study assessed changes in body composition and 24‐h energy metabolism at 6 and 12 months after initiation of a 1‐month very low‐calorie ketogenic diet (VLCKD) in women with obesity.

**Methods:**

Seventeen women with obesity who completed a 1‐month VLCKD underwent a 4‐week transition phase with carbohydrate reintroduction, followed by a hypocaloric balanced diet. Assessments of body composition by dual‐energy X‐ray absorptiometry (DXA) and 24‐h energy expenditure (24hEE) by a whole‐room indirect calorimeter were performed.

**Results:**

Following the initial 7% weight loss, body weight further decreased at 6 months (−3.9%, *p* < 0.05), primarily driven by a significant decrease in fat mass (−10%, *p* < 0.05). From 6 to 12 months, three participants continued to lose weight, whereas most remained stable or partially regained. Lean soft tissue, decreased during the VLCKD phase, remained stable throughout follow‐up. Both 24hEE and 24‐h sleeping metabolic rate exhibited a progressive trend toward increase. Minute‐by‐minute 24hEE trajectories revealed a significant increase in metabolic rate from 1 to 6 months (*p* < 0.001). The metabolic adaptation observed after 1 month of VLCKD was no longer detectable at either 6 or 12 months.

**Conclusions:**

These findings provide novel insight into the physiological adaptations following VLCKD, supporting its role in supervised weight loss programs for selected patients.

**Trial Registration:**

ClinicalTrials.gov identifier: NCT07418281

## 1 Introduction

Obesity is a chronic disease marked by excess adiposity and represents a major public health concern [1]. Calorie‐restricted nutrition and lifestyle modification represent the first‐line therapeutic approach, preceding pharmacologic or surgical interventions [2, 3]. Among dietary interventions, the very low‐calorie ketogenic diet (VLCKD) has emerged as an effective weight loss strategy [4, 5, 6, 7, 8], with the potential advantage of preserving lean body mass [4, 9, 10], the main determinant of energy expenditure [11, 12, 13].

In a previous study [14] we showed that, after 1 month of VLCKD, females with obesity lost 7% of their initial body weight, which reflected an 8.8% reduction in fat mass (FM) and a 5.6% decrease in lean soft tissue (LST). These early changes were accompanied by substantial shifts in substrate utilization, notably a marked decrease in carbohydrate oxidation and a pronounced increase in fat oxidation. Importantly, we also observed a 10% reduction in 24‐h energy expenditure (24hEE) and sleeping metabolic rate (24hSMR). These observations highlight a critical need to explore how the trajectory of body composition and energy metabolism may change following completion of the VLCKD course [8].

Emerging evidence from randomized trials and long‐term observational studies suggests that, when followed by structured dietary reintroduction and maintenance programs, a substantial proportion of this weight loss, largely driven by reductions in FM, can be sustained over the subsequent 6–12 months [15, 16, 17, 18, 19]. Compared to low‐calorie balanced diets, the VLCKD induced more favorable changes in body composition, with relative preservation of lean mass [16, 18].

To date, the medium‐ and long‐term trajectory of 24hEE following VLCKD remains unknown, particularly when assessed using whole‐room indirect calorimetry, the gold‐standard method for measuring energy metabolism. This represents a critical gap, as it remains unclear whether the early reduction in energy expenditure reflects a transient adaptive response or persists over time, with important clinical implications given that sustained reductions in energy expenditure may promote weight regain. Therefore, this study aimed at evaluating the changes in 24hEE and macronutrient oxidation, as assessed by gold‐standard whole‐room indirect calorimetry, and in body composition assessed by dual‐energy X‐ray absorptiometry (DXA) at 6 and 12 months in women with obesity who completed the initial 1‐month VLCKD phase. Given the lack of long‐term studies assessing 24hEE following VLCKD, we hypothesized, as a preliminary assumption, that the short‐term reduction in energy expenditure observed during the 1‐month active phase [14] could represent a transient adaptive response to caloric restriction, potentially occurring alongside changes in body composition and with the possible involvement of thyroid hormones.

## 2 Methods

### 2.1 Patients and Study Design

This prospective longitudinal follow‐up study included 17 adult women with obesity (BMI > 30 kg/m^2^) who had previously completed a 1‐month VLCKD at the Obesity and Lipodystrophy Center, University Hospital of Pisa [14]. Recruitment procedures and the VLCKD protocol have been already described in detail [14]. Briefly, participants followed a 1‐month VLCKD period (700–800 kcal/day; 11% carbohydrate, 46% fat, 43% protein) and underwent 24‐h metabolic assessments in a whole‐room indirect calorimeter at baseline (V1, Day 1), after 1 week (V2, Day 8), and after 4 weeks (V3, Day 29), while consuming the same standardized VLCKD throughout [14]. After completing the VLCKD, participants entered a 4‐week structured dietary reintroduction phase under dietitian supervision, with weekly phone‐call visits during which carbohydrate intake was progressively increased to a balanced hypocaloric diet. Subsequently, they followed a supervised program that included individualized meal plans, lifestyle reinforcement, and monthly nutritional counseling sessions. Follow‐up visits were conducted at 6 (V4) and 12 (V5) months from V1, during which participants underwent assessment of body composition and 24hEE. A total of 14 participants returned at V4 (1 underwent bariatric surgery, 2 discontinued for personal reasons), and 11 participants returned at V5 (1 restarted VLCKD, 2 discontinued for personal reasons).

All participants provided written informed consent, and the study protocol was approved by the Local Ethical Review Board (CEAVNO—Comitato Etico Area Vasta Nord Ovest, serial number 19543). The study was conducted in accordance with the Declaration of Helsinki and its later amendments.

In the present follow‐up study, participants underwent 24‐h whole‐room indirect calorimetry at V4 and V5, and fasting body composition was reassessed by DXA at the end of each calorimetry session, following the same procedures used at V1 and V3 [14]. At each visit, body weight was measured using a calibrated digital scale with participants wearing light clothing. Standing height was measured without shoes to the nearest 0.1 cm using a stadiometer, and body mass index (BMI) was calculated as weight in kilograms divided by height in meters squared. At each visit, fasting blood samples were collected at 7:30 a.m. before entering the metabolic chamber.

Among the 14 participants who were seen at the 6‐month follow‐up, 2 were receiving hypolipidemic therapy, 2 were on vitamin D supplementation, 1 was treated with metformin combined with antihypertensive and hypolipidemic agents, and 2 were on L‐thyroxine replacement for autoimmune hypothyroidism (all with normal thyrotropin [TSH] levels). Among the 11 patients who returned at 12 months, 2 were on hypolipidemic therapy, 1 continued metformin with antihypertensive and hypolipidemic treatment, and 2 remained on L‐thyroxine with normal TSH.

### 2.2 Dietary Intervention

**FIGURE 1:**
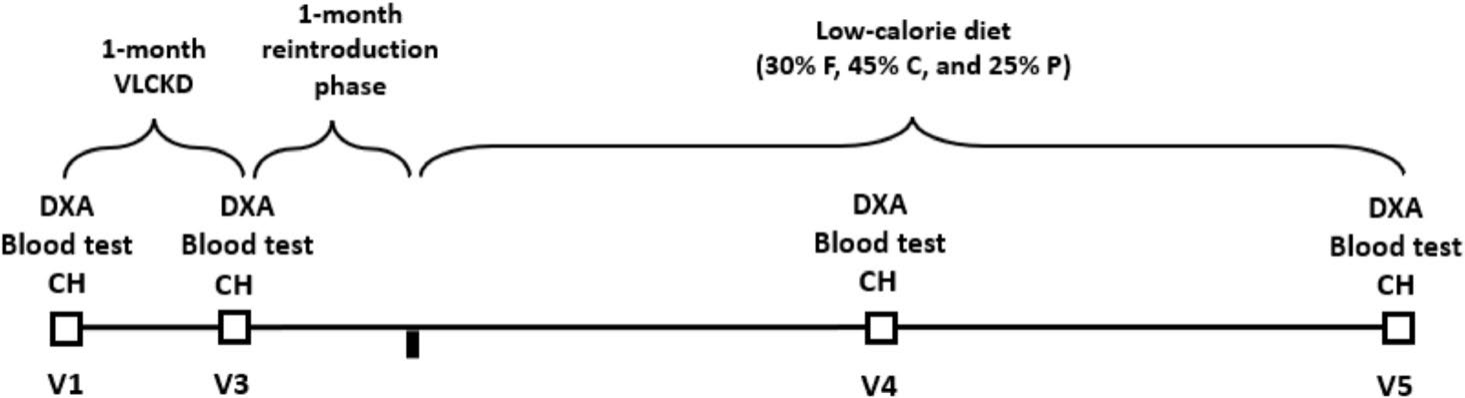
V1–V3: Active phase of VLCKD consisting of a very low‐calorie diet (700–800 kcal/day), low in carbohydrate (< 30 g/day) and fat (only 20 g/day) with protein ranging between 1.2 and 1.5 g per kilogram of ideal body weight. V1: CH for assessing the 24‐h energy expenditure on the first day of VLCKD, blood test, and DXA after an overnight fast after exiting the metabolic chamber. V3: CH for assessing the 24‐h energy expenditure after 1‐month time frame from V1, blood test, and DXA after an overnight fast after exiting the metabolic chamber. V4: CH for assessing the 24‐h energy expenditure 6 months from V1, blood test, and DXA after an overnight fast after exiting the metabolic chamber. V5: CH for assessing the 24‐h energy expenditure 12 months from V1, blood test, and DXA after an overnight fast after exiting the metabolic chamber. CH, whole‐room indirect calorimetry, C, carbohydrate; DXA, dual‐energy X‐ray absorptiometry; F, fat; P, protein.

After the initial 1‐month VLCKD phase, a subsequent transition period (4 weeks) was implemented, during which carbohydrates were gradually reintroduced. The process started with the addition of foods with a low glycemic index (fruit, 1 week), followed by foods with a moderate glycemic index (legumes and low‐fat dairy products, 1 week), and finally foods with a high glycemic index (bread, pasta, and cereals) during the subsequent 2 weeks, with energy intake progressively increasing from 800 to 1400 kcal/day. The diet was tailored to each patient based on individual preferences. After the 1‐month reintroduction phase, a transition to a hypocaloric Mediterranean diet was completed (30% fat, 45% carbohydrate, and 25% protein). Total energy intake was individualized according to age, body weight, height, and physical activity level, resulting in approximately 25 kcal per kilogram of ideal body weight. Patients were encouraged to engage in regular physical activity. This regimen continued throughout the follow‐up study period, during which participants underwent body composition and 24‐h energy metabolism assessments at V4 and V5. During the 24‐h stay in the metabolic chamber (V4 and V5), participants were provided with their diet, reduced by 20% to account for the lower physical activity inside the calorimeter. Meals were served at standardized times: breakfast at 8:00 a.m., lunch at 12:00 p.m., and dinner at 7:00 p.m., providing 20% of total daily calories at breakfast, 50% at lunch, and 30% at dinner. The study design is presented in Figure 1.

### 2.3 Whole‐Room Indirect Calorimetry

Assessment of 24hEE and respiratory exchange ratio (RER), an indicator of substrate oxidation, was conducted within a whole‐room indirect calorimeter following established protocols [20, 21, 22]. Following an overnight fast, participants entered the calorimeter at approximately 7:30 a.m. The weight of any unconsumed food was recorded to determine the actual 24‐h energy intake during each session. Patients and controls remained in the calorimeter for 23.5 h, during which carbon dioxide (CO_2_) production and oxygen (O_2_) consumption were recorded every minute. The 24hEE was calculated using the Lusk equation [11]. The chamber air temperature was maintained at 23.8°C ± 1.4°C, and the air inflow rate was regulated by a mass flow controller at a constant rate of 100 L/min. Air samples from the inlet pipe and chamber were drawn by membrane pumps, dried to a humidity level below 1000 ppm using a gas sample dryer driven by counterflow dry medical air, and subsequently analyzed using absolute gas analyzers. Spontaneous physical activity was assessed using radar sensors and expressed as the percentage of time during which activity was detected [23]. Calorimeter accuracy was verified before and during the study by monthly combustion of instrument‐grade propane on a calibrated scale. The recovery rates for O_2_ consumption, CO_2_ production, and RER were maintained within 5% of values predicted by stoichiometric calculations. Daytime energy expenditure in the inactive, awake state (EE_0_) was obtained as the intercept of the regression line relating EE to spontaneous physical activity, measured at 1‐min intervals between 10:00 a.m. and 1:00 a.m., and extrapolated to 15 h [24]. Sleeping energy expenditure (Sleep) was determined as the average EE recorded between 11:30 p.m. and 5:00 a.m., restricted to periods with movement < 1.5% (< 0.9 s/min), and extrapolated to 24 h. Awake and fed thermogenesis was calculated as the difference between daytime resting EE (EE_0_) and Sleep (kcal/min) and extrapolated to a 15‐h waking period. The 24hRER was determined as the ratio of 24‐h mean CO_2_ production to O_2_ consumption. Carbohydrate (CarbOx) and lipid (FatOx) oxidation rates were calculated from the nonprotein 24hRER (NPRER) and nonprotein oxygen consumption (NPVO_2_), after correction for protein oxidation (ProtOx), which was estimated based on 24‐h urinary nitrogen excretion [25], as follows:

$$\begin{array}{c} \text{Carbohydrate oxidation rate}\;\left(\text{kcal}/\mathrm{day}\right)\\ \;={\text{NPVO}}_{2}\cdot \left[\left(\text{NPRER}-0.707\right)/0.293\right]\cdot 5.047 \end{array}$$

$$\text{Lipid oxidation rate}\;\left(\text{kcal}/\mathrm{day}\right)={\text{NPVO}}_{2}\cdot \left[\left(1-\text{NPRER}\right)/0.293\right]\cdot 4.686$$

Nitrogen excretion was assessed using the UREAL assay (Cobas c, Roche Diagnostics), and urinary nitrogen was calculated from urinary urea concentration using the following formula: urinary nitrogen = urea × 0.467.

### 2.4 Measurement of Body Composition

Body composition was assessed using DXA (Lunar Prodigy, GE HealthCare): The analysis provided quantitative estimates of several components [26], including LST, defined as the total mass of non‐fat and non‐bone mineral tissues, and FM. Additionally, appendicular lean soft tissue (ALST) and appendicular fat mass (AFM) were calculated as the sum of the LST and FM of both upper and lower limbs, respectively. Trunk fat mass (TFM) was derived by subtracting AFM from total FM.

### 2.5 Assays for Serum Hormones and Metabolic Analytes

Serum concentrations of thyrotropin (TSH), free triiodothyronine (FT3), and free thyroxine (FT4) were determined using a highly specific and sensitive automated chemiluminescent immunoassay (CLIA) (Vitros 3600 System, Ortho‐Clinical Diagnostics). Reference ranges were 0.4–4.0 mUI/L for TSH, 7–17 pg/mL for FT4, and 2.7–5.7 pg/mL for FT3. Additional biochemical analyses included serum insulin, creatinine, total cholesterol, LDL, HDL, triglycerides, and liver enzymes, all quantified using standardized commercial assay kits.

### 2.6 Statistical Analysis

All statistical analyses were performed using SAS software, version 9.2 (SAS Institute Inc.). Variables with a normal distribution were summarized as mean ± standard deviation (SD). A *p* value < 0.05 was considered statistically significant. TSH concentrations were log10‐transformed prior to analysis to improve normality of the distribution.

Statistical analyses were performed using two complementary approaches. Mixed‐effects repeated measures models were fitted to assess trajectories of body composition and energy metabolism parameters across all study visits, utilizing all available data at each time point (V1: *n* = 17, V3: *n* = 17, V4: *n* = 14, V5: *n* = 11). These models included fixed effects for visits and random effects for participants, accounting for within‐subject correlation using a first‐order autoregressive covariance structure. Least‐squares (LS) means with 95% confidence intervals (CI) were estimated from these models and compared using least significant difference (LSD) post hoc test for pairwise comparisons between all visits. Additionally, paired comparisons using paired *t*‐tests were conducted for participants who completed specific follow‐up assessments to evaluate within‐subject changes in those with complete data (14 participants at V4, 11 participants at V5). Results from paired analyses are reported as mean changes with percentages.

Energy expenditure trajectories were analyzed using minute‐by‐minute data collected during the 24‐h metabolic chamber sessions. Linear mixed‐effects models compared EE profiles between V3 and V4 with fixed effects for time point, time of day, and their interaction, plus random intercepts and slopes by participant. Separate analyses examined the nighttime period (11:30 p.m. to 5:00 a.m.). Models were fit using restricted maximum likelihood.

Metabolic adaptation was assessed using 24hSMR. Predicted 24hSMR was estimated from a linear regression model (*R* ^2^ adj = 0.79, *p* < 0.001) derived from an independent cohort of 22 healthy women of comparable age and body weight, consuming a weight‐maintaining eucaloric diet during a standardized calorimetry session. No data from VLCKD participants, including baseline data, were used to derive or calibrate the prediction equation. Metabolic adaptation was computed at the individual level as observed minus predicted 24hSMR. To minimize the potential confounding effect of preexisting interindividual variability in thermogenic efficiency, a baseline‐residual‐corrected estimate was additionally computed as a sensitivity analysis following the approach of Galgani and Santos [27]: baseline‐corrected MA (metabolic adaption) = (observed − predicted 24hSMR) at Vk − (observed − predicted 24hSMR) at V1, where Vk indicates the specific follow‐up visit. The use of 24hSMR rather than total 24hEE for the adaptation analysis was deliberate, as 24hSMR is measured under controlled nocturnal conditions and is less influenced by variations in physical activity, energy intake, and diet‐induced thermogenesis.

## 3 Results

### 3.1 Changes in Body Composition

**FIGURE 2:**
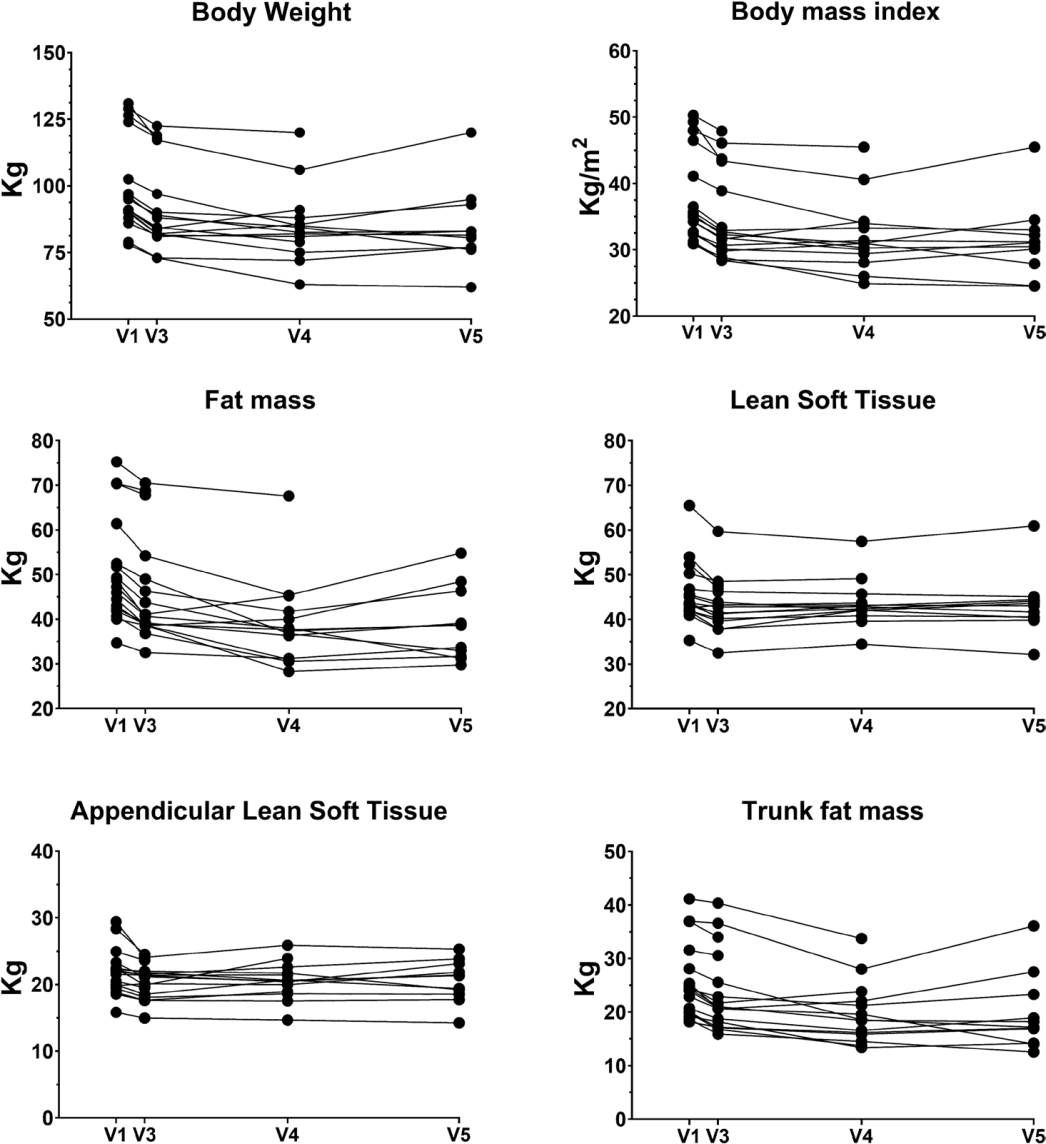
Individual body weight and body composition parameters assessed by dual‐energy X‐ray absorptiometry (DXA) at each visit. V1 and V3: *N* = 17; V4: *N* = 14; V5: *N* = 11.

On average, among the 14 patients who completed the 6‐month follow‐up, an additional 3.9% reduction in body weight was observed from V3 to V4, driven by a 10% reduction in FM. In the 11 patients who completed the 1‐year follow‐up, a modest increase of 2.7% in body weight, with a 6.7% rise in total FM, was observed from V4 to V5. At variance, LST that was reduced during the VLCKD phase did not show significant changes over the follow‐up period from V3 to V5, though a tendency to increase was observed. At 12 months, loss of LST accounted for ~20% of total weight loss. Of note, among the 11 patients who completed the 12‐month follow‐up, 3 participants showed an additional decrease in body weight, whereas the majority either remained stable or showed a partial recovery (Figure 2).

To compare changes in body composition parameters across study visits, results were expressed as LS means (95% CI) and analyzed using mixed‐effects repeated measures models (Table 1).

**TABLE 1:** Body composition measures of the study population.

	V1	V3	V4	V5
BW (kg)	99.1 (90.6; 107.7)	92.1* (83.5; 100.6)	87.9* ^#^ (79.3; 96.5)	90.0* (81.3; 98.7)
BMI (kg/m^2^)	37.5 (34.1; 40.9)	34.7* (31.4; 38.2)	33.6* ^#^ (30.1; 37.0)	33.9* (30.5; 37.4)
FM (kg)	50.6 (44.6; 56.7)	46.2* (40.3; 52.3)	41.8* ^#^ (35.7; 47.9)	43.5* (37.3; 49.7)
LST (kg)	45.8 (42.7; 48.9)	43.1* (40.0; 46.3)	43.9* (40.7; 47.0)	44.0* (40.8; 47.2)
ALST (kg)	21.9 (20.3; 23.4)	20.5* (18.9; 22.0)	20.9 (19.4; 22.5)	21.1 (19.5; 22.7)
TFM (kg)	25.7 (22.2; 29.3)	23.4* (19.9; 27.0)	20.7* ^#^ (17.2; 24.2)	21.6* (18.0; 25.1)
AFM (kg)	23.9 (20.8; 27.0)	21.7* (18.5; 24.8)	19.9* ^#^ (16.8; 23.1)	20.7* (17.5; 24.0)

*Note:* Each parameter in the table is expressed as least‐squares means (95% CI) and was compared using LSD post hoc test for pairwise comparisons between visits.

Abbreviations: AFM, appendicular fat mass; ALST, appendicular lean soft tissue; BW, body weight; FM, fat mass; LST, lean soft tissue; TFM, trunk fat mass.

* *p* < 0.05 versus V1 by LSD post hoc analysis.

^#^ *p* < 0.05 versus V3 by LSD post hoc analysis.

### 3.2 Assessment of Energy Metabolism

Measures of energy intake along with metabolic measures are reported in Table 2.

**TABLE 2:** Energy intake and metabolic measures of the study population.

	V1	V3	V4	V5
Energy and macronutrient intake				
Prescribed energy intake (kcal/day)	761.4 ± 29.2	768.0 ± 25.4	1665.5 ± 176.8	1670.8 ± 176.8
Prescribed energy intake (kcal/kg BW)	7.86 ± 1.12	8.53 ± 1.31	19.7 ± 2.03	20.0 ± 2.06
Actual energy intake (kcal/day)	740.8 ± 40.1	680.7 ± 114.4	1600.4 ± 190.9	1620.6 ± 248.6
Actual energy intake (kcal/kg BW)	7.62 ± 1.11	7.54 ± 1.59	18.9 ± 2.2	19.3 ± 1.6
CHO intake (kcal/day)	79.8 ± 8.7	72.3 ± 19.4	792.2 ± 106.5	806.7 ± 124.4
CHO intake (kcal/kg BW)	0.8 ± 0.14	0.8 ± 0.24	9.4 ± 1.24	9.6 ± 0.90
Fat intake (kcal/day)	339.9 ± 52.1	317.4 ± 75.2	490.1 ± 57.6	488.1 ± 76.8
Fat intake (kcal/kg BW)	3.5 ± 0.71	3.5 ± 0.93	5.8 ± 0.63	5.8 ± 0.43
PRO intake (kcal/day)	313.3 ± 43.2	295.5 ± 55.4	318.1 ± 35.8	325.8 ± 51.7
PRO intake (kcal/kg BW)	3.2 ± 0.62	3.3 ± 0.75	3.8 ± 0.43	3.9 ± 0.43
Metabolic measures				
Measured 24hEE (kcal/day)	1746 ± 336	1594 ± 196	1650 ± 190	1673 ± 230
Predicted 24hEE (kcal/day)	1826 ± 134	1773 ± 117	1780 ± 102	1776 ± 136
Metabolic adaptation 24hEE	−79.8 ± 244.8	−179.7 ± 160.2	−129.2 ± 166.8	−101.8 ± 129.4
V1‐corrected Metabolic adaptation 24hEE	0 ± 0	−76.3 ± 173.8	22.3 ± 306.4	40.9 ± 112.6
Measured 24hSMR (kcal/day)	1297 ± 291	1165 ± 291	1256 ± 230	1217 ± 212
Predicted 24hSMR (kcal/day)	1329 ± 144	1273 ± 125	1280 ± 109	1275 ± 146
Metabolic adaptation 24hSMR	−31.7 ± 213.4	−108.0 ± 175.4	−24.1 ± 215.7	−58.2 ± 127.7
V1‐corrected Metabolic adaptation 24hSMR	0 ± 0	−99.8 ± 212.9	−3.3 ± 307.6	95.4 ± 146.9
24hRER (ratio)	0.789 ± 0.05	0.752 ± 0.05	0.842 ± 0.03	0.841 ± 0.02
CarbOx (kcal/day)	371.9 ± 293.2	129.2 ± 243.5	662.3 ± 185.9	650.1 ± 121.5
FatOx (kcal/day)	999.5 ± 436.7	1104.8 ± 387.4	626.9 ± 194.2	641.9 ± 183.3
ProtOx (kcal/day)	349.6 ± 70.5	321.9 ± 43.7	337.5 ± 127.0	365.5 ± 100.8

*Note:* Prescribed total energy intake (kcal/day) refers to the amount of energy allocated to patients following VLCKD during each session (V1, V3, V4, V5) in the metabolic chamber. Actual total energy intake represents the energy effectively consumed by patients, accounting for any unconsumed food during each session in the metabolic chamber. Macronutrient intake refers to actual intake. Conventional metabolic adaptation was calculated as observed minus predicted 24hEE and 24hSMR. V1‐corrected metabolic adaptation was calculated as the follow‐up residual minus the baseline residual, according to the approach of Galgani and Santos [27]. Data are presented as mean ± SD. V1: First day of VLCKD; V3: Day 29 of VLCKD; V4: 6 months from V1; V5: 12 months from V1.

Abbreviations: BW, body weight; CarbOx, carbohydrate oxidation; CHO, carbohydrate; FatOx, fat oxidation; Pro, protein; ProtOx, protein oxidation.

**FIGURE 3:**
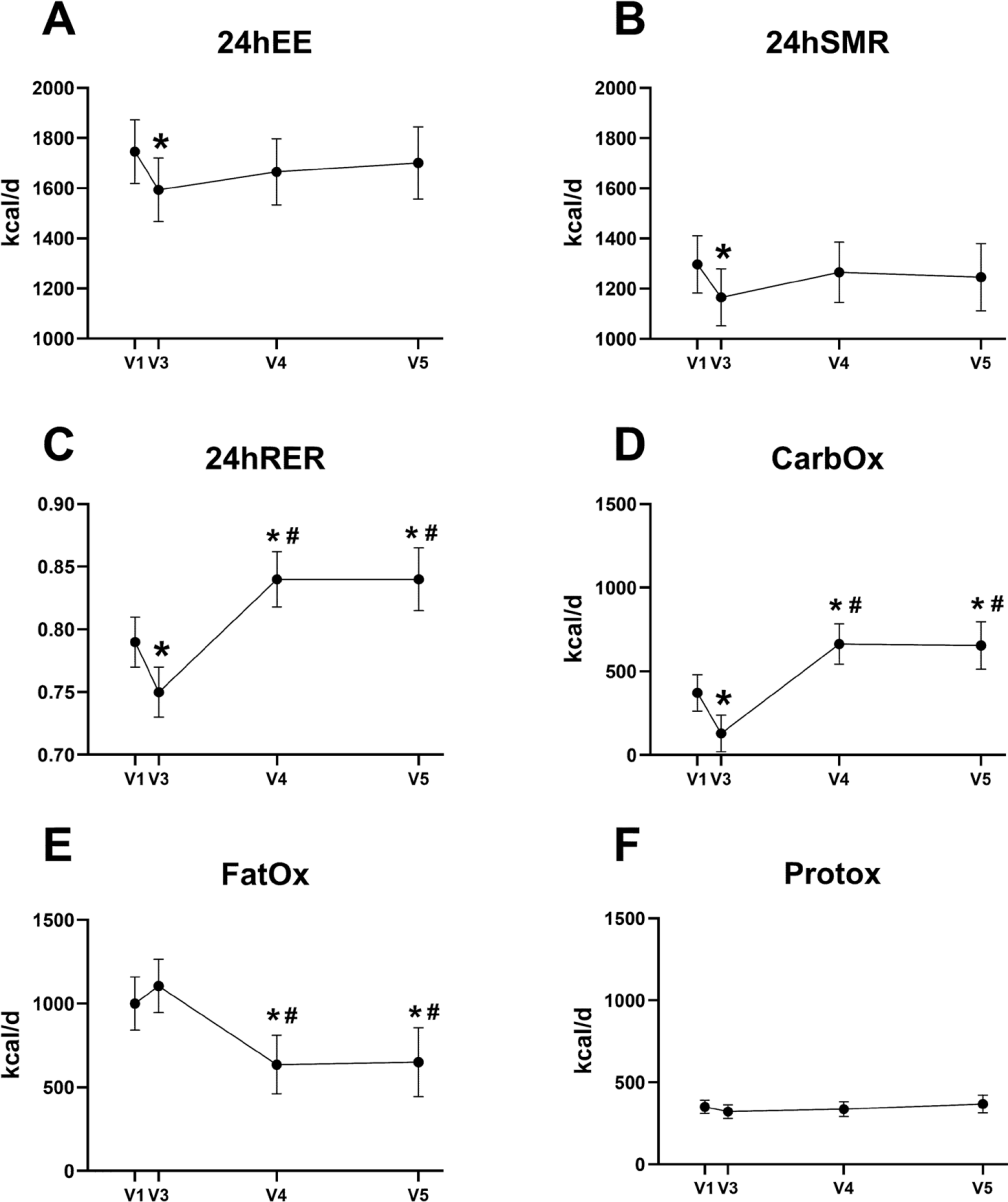
(A) 24‐h energy expenditure (24hEE), (B) sleeping metabolic rate (24hSMR), (C) respiratory exchange ratio (24hRER), (D) carbohydrate oxidation, (E) fat oxidation, and (F) protein oxidation across study visits. Data are expressed as least‐squares means with 95% CI (error bars) derived from mixed‐effects repeated measures models. Statistical comparisons were performed using LSD post hoc test for pairwise comparisons between visits. *p < 0.05 versus V1 by LSD post hoc analysis. ^#^ *p* < 0.05 versus V3. V1: First day of VLCKD; V3: Day 29 of VLCKD; V4: 6 months from V1; V5: 12 months from V1.

Results of 24hEE and macronutrient oxidation measurements are shown in Figure 3, expressed as LS means with 95% confidence intervals from mixed‐effects repeated measures models. From V3 to V5, both 24hEE (Figure 3A) and 24hSMR (Figure 3B) showed a trend toward an increase, though not reaching statistical significance (*p* = 0.177 and *p* = 0.079 vs. V3, respectively). In contrast, 24hRER (Figure 3C) exhibited a marked rise from V3 to V4, which was sustained through V5 (*p* < 0.001 for both comparisons vs. V3). This increase was driven by a significant increase in carbohydrate oxidation (Figure 3D, *p* < 0.001 vs. V3) and a concomitant reduction in fat oxidation (Figure 3E, *p* < 0.001 vs. V3), while protein oxidation remained unchanged (Figure 3F).

**FIGURE 4:**
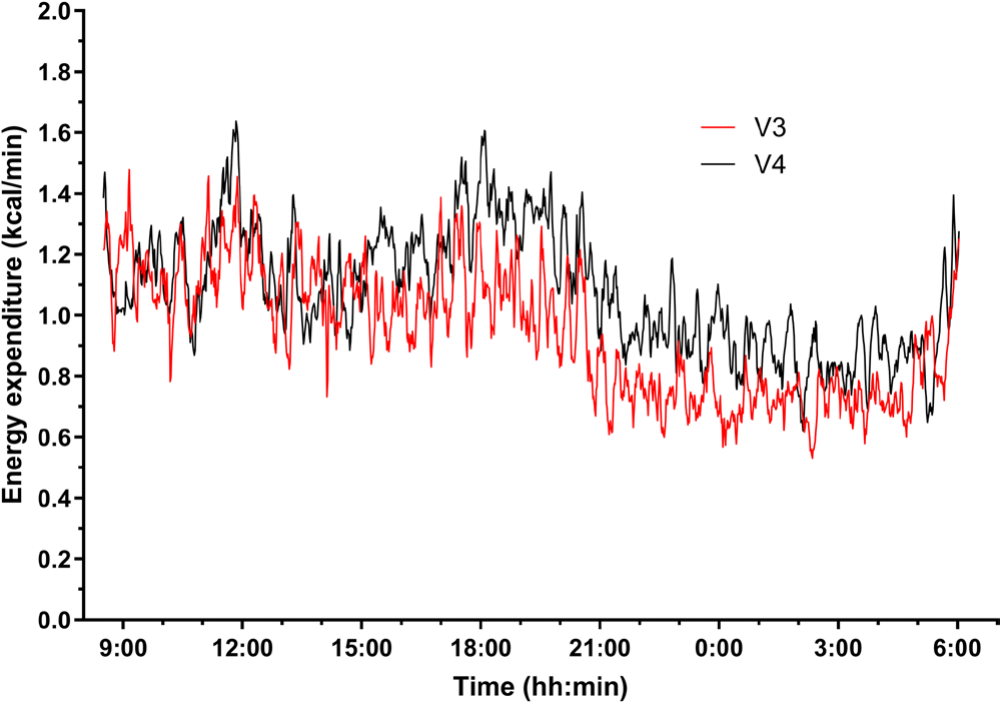
Median minute‐by‐minute energy expenditure (kcal/min) at V3 (1 month, red line) and V4 (6 months, black line) in 14 participants with valid chamber data. Mixed‐effects model analysis revealed a significant increase in EE from V3 to V4 (*β* = 0.072 kcal/min, *p* < 0.001), equivalent to 104 kcal/day. The recovery was particularly pronounced during the nighttime period (0.239 kcal/min, *p* < 0.001), accounting for 76% of the total daily increase. [Color figure can be viewed at wileyonlinelibrary.com]

Figure 4 presents the average minute‐by‐minute EE profiles over the 24‐h period at V3 (end of VLCKD) and V4 (6 months) in the 14 participants who completed both visits. The increase in energy expenditure at V4 was partially explained by higher caloric intake, which increased the thermic effect of food. However, recovery was also evident during the sleeping period, with a significant nighttime‐specific increase of 0.239 kcal/min (*p* < 0.001), representing 76% of the total daily recovery in EE.

Metabolic adaptation was assessed by comparing observed to predicted 24hSMR at each visit (Table 2). At baseline, no adaptation was present (*p* = 0.549). Following 1 month of VLCKD, observed 24hSMR (1165 kcal/day) was significantly lower than predicted (1273 kcal/day), indicating metabolic adaptation (mean difference: −108 kcal/day, 95% CI: −198 to −18, *p* = 0.022). This adaptation was abolished at 6 months (*p* = 0.683) and remained nonsignificant at 12 months (*p* = 0.161). In the baseline‐residual‐corrected sensitivity analysis, the V3 estimate was directionally similar but did not reach statistical significance (−76.3 ± 173.8 kcal/day, 95% CI: −165.6 to 13.0, *p* = 0.089). At V4 and V5, neither the conventional nor the baseline‐corrected approach showed significant metabolic adaptation, supporting the absence of sustained metabolic adaptation during follow‐up.

### 3.3 Changes in Serum Hormones and Other Blood Parameters

**FIGURE 5:**
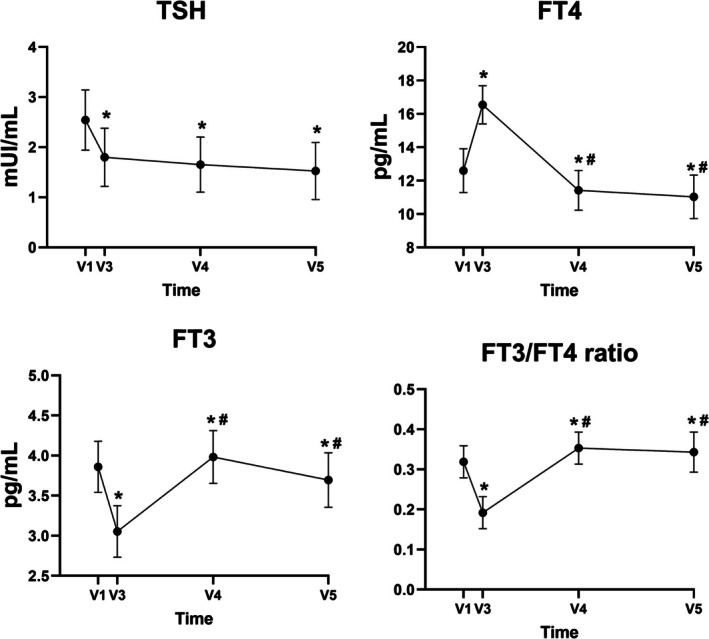
Serum thyrotropin (TSH), free T4, free T3, and free T4/free T3 ratio concentrations at baseline (V1), end of VLCKD (V3), and follow‐up visits (V4, V5). Data are expressed as least‐squares means with 95% CI (error bars) derived from mixed‐effects repeated measures models. Fasting blood samples were collected at 7:30 a.m. before entering the metabolic chamber at each visit. Statistical comparisons were performed using LSD post hoc test for pairwise comparisons between visits. Values at V1 correspond to pre‐V1 of the previous study [14] before initiation of VLCKD. *p < 0.05 versus V1. #*p* < 0.05 versus V3. V1: First day of VLCKD; V3: 1 month after V1; V4: 6 months after V1; V5: 12 months after V1.

As previously reported [14], the 1‐month VLCKD was associated with a significant increase in FT4 and decreases in FT3 and serum TSH. Following the transition to a balanced hypocaloric diet, a marked reversal of these changes was observed (Figure 5). From V3 to V4, FT4 decreased significantly (*p* < 0.001 vs. V3) while FT3 increased significantly (*p* < 0.001 vs. V3), with both hormones approaching baseline values. At V5, FT4 remained stable (*p* = 0.45 vs. V4), while FT3 showed a modest but significant decrease compared to V4 (*p* = 0.031), though remaining higher than V3 levels (*p* < 0.001 vs. V3) and not significantly different from baseline (*p* = 0.37 vs. V1). TSH levels showed no significant changes from V3 through V5 (*p* = 0.46 at V4 and *p* = 0.27 at V5 vs. V3). The temporal pattern of thyroid hormone recovery paralleled the restoration of energy expenditure, with both processes largely complete by 6 months.

Regarding lipid metabolism, mixed‐model analysis revealed significant increases in LDL cholesterol (*p* = 0.01), HDL cholesterol (*p* = 0.03), and triglycerides (*p* = 0.043) at V4 compared to V3. At V5, HDL cholesterol remained significantly elevated compared to V3 (*p* = 0.034), while LDL cholesterol (*p* = 0.15) and triglycerides (*p* = 0.31) were no longer significantly different from V3 levels, reflecting the transition from ketogenic to carbohydrate‐inclusive metabolism. Fasting insulin levels showed no significant changes from V3 to V4 or V5 (*p* = 0.120 and *p* = 0.760, respectively).

## 4 Discussion

In this study, we explored the preliminary hypothesis that short‐term reductions in energy expenditure after a 1‐month VLCKD could reflect a transient adaptive response to caloric restriction, potentially occurring alongside changes in body composition. To investigate this, we examined the effects of a 1‐month VLCKD followed by 11 months of a hypocaloric balanced diet on body composition and 24‐h energy metabolism, assessed by gold‐standard whole‐room indirect calorimetry.

First, women with obesity who achieved a reduction in body weight during a 1‐month VLCKD exhibited further weight loss during a hypocaloric balanced diet up to 6 months, with maintenance of the achieved results or partial recovery over the subsequent 6 months.

Previous evidence indicates that a VLCKD promotes favorable changes in body weight and composition over both the medium and long term [5]. In patients with overweight or obesity, a VLCKD has shown greater weight loss and more favorable body composition changes than low‐calorie diets over a follow‐up period of up to 24 months, with marked fat mass (FM) reduction and relatively better preservation of fat‐free mass [16, 17, 18].

In our previous study [14], we showed that after 1 month of VLCKD participants lost approximately 7% of their initial body weight, corresponding to an 8.8% reduction in FM. At the same time, a smaller yet meaningful 5.6% decline in LST was observed, accounting for 38% of the total weight loss. The results of the present study show that the substantial weight loss achieved during the initial VLCKD month was augmented by 6 months and largely maintained over 12 months. Over the entire study period, weight loss was driven predominantly by reductions in total FM. The findings of the present study support the notion that, when accompanied by structured food reintroduction and a supervised dietary and physical activity program, loss of FM, particularly TFM as assessed by DXA, can be maintained over the long term with minor impact on LST. Of note, body composition analyses were limited to participants completing on‐site follow‐up (14 at 6 months; 11 at 12 months), due to incomplete follow‐up in a few patients.

In previous clinical trials evaluating GLP‐1–based obesity therapies, the lean mass loss relative to total weight loss varied widely, ranging from approximately 15% to 39% across different drugs, including liraglutide, semaglutide, tirzepatide, and retatrutide [28, 29, 30, 31], albeit across heterogeneous clinical settings, study designs, and treatment durations.

Within this context, the VLCKD may represent a therapeutic dietary approach that may be particularly beneficial for individuals requiring weight reduction within a defined time frame, such as in the preoperative setting to reduce surgical risk in patients undergoing elective procedures, including bariatric surgery, where preoperative weight loss is associated with improved surgical feasibility.

Understanding the potential metabolic changes following dietary interventions is crucial, as sustained reductions in energy expenditure are thought to contribute to weight regain and may differ according to dietary modality and the degree of lean mass preservation [32].

To our knowledge, no studies have assessed the long‐term effects of VLCKD on 24‐h energy metabolism using a precise and accurate method such as whole‐room indirect calorimetry [8].

In our previous study, we demonstrated a clear‐cut decrease in 24EE and 24hSMR after 1 month of VLCKD [14]. In the present follow‐up, we observed stabilization in both measures at 6 months, despite further reduction of body weight, without substantial changes over the 12‐month period. The concurrent rise in RER driven by a marked increase in carbohydrate oxidation and a reduction in fat oxidation reflects a gradual shift from the ketogenic metabolic profile to a balanced fuel oxidation pattern, as dietary reintroduction progresses.

The recovery in energy expenditure was at least partially explained by the progressive increase in caloric intake during the refeeding phase; however, this phenomenon was mainly observed for 24hSMR. In response to weight loss and negative energy balance, resting metabolic rate often declines to a greater extent than predicted by reductions in LST and FM, a phenomenon commonly referred to as metabolic adaptation [32]. This adaptive response is widely recognized as a key mechanism underlying resistance to further weight loss and the propensity for weight regain [32, 33, 34, 35]. Notably, our findings indicate that the metabolic adaptation observed during the active VLCKD phase, characterized by marked caloric restriction and negative energy balance, was no longer evident after the refeeding phase. This reversal may be mediated by multiple mechanisms, including the potential modulation of metabolic efficiency regulators such as thyroid hormones. Thyroid hormones are key regulators of thermogenesis and energy expenditure via multiple complementary mechanisms [36], including stimulation of energetically costly substrate cycling (glycolysis/gluconeogenesis, lipolysis/lipogenesis, and protein turnover), increased transmembrane ion leakage with higher ATP turnover, promotion of nongenomic actions and mitochondrial biogenesis, and activation of uncoupling processes that reduce the efficiency of ATP synthesis in favor of heat dissipation. Thyroid hormone–induced increases in heart rate further contribute to elevated whole‐body energy expenditure. REE is very sensitive to minor changes of TSH [37], and the intraindividual changes in REE correlated significantly with the corresponding changes in FT4 [38]. Previous studies support a central role for thyroid hormones in adaptive thermogenesis, whereby sympathetic activation promotes type II iodothyronine deiodinase activity in brown adipose tissue [39, 40], increasing intracellular conversion of T4 to T3 and thereby contributing to adaptive thermogenic responses. Results of the present study suggest that during follow‐up, the rise in T3 levels, whose conversion from T4 is suppressed during caloric deprivation [20, 41, 42], may contribute to the observed increase in 24hEE and 24hSMR by enhancing metabolic rate. However, no significant associations were observed between changes in FT3 or FT4 and metabolic adaptation, possibly due to the limited sample size. Therefore, while the temporal pattern of thyroid hormone changes paralleled the recovery of energy expenditure, a direct mechanistic link cannot be established based on the current data. The involvement of thyroid hormones remains a plausible but unproven contributor and warrants further investigation in larger studies. Serum TSH concentrations are higher in individuals with obesity compared to normal‐weight participants [43]. The reduction in serum TSH observed in the present study is likely attributable to weight loss induced by VLCKD.

As previously reported [14], no adverse events occurred during the active phase of the VLCKD intervention, and this safety profile was consistently maintained throughout the follow‐up period. These findings further support the notion that, within a supervised weight maintenance program, a VLCKD represents a safe and well tolerated nutritional strategy in this clinical context.

This study has several limitations. First, the sample size was relatively small, reflecting the complexity and resource‐intensive nature of whole‐room indirect calorimetry, which limits the feasibility of large‐scale studies. Although this limited sample size may reduce generalizability, the consistency of the observed changes across time points supports the robustness of the reported pattern of sustained FM reduction with minimal impact on LST among completers. Second, although the study population consisted exclusively of women with obesity, some degree of clinical heterogeneity was present, including differences in comorbidities and concomitant treatments. These factors may limit the generalizability of the findings to broader populations, including men or individuals with different metabolic profiles. Participant attrition over the 12‐month follow‐up reduced statistical power and may have introduced selection bias, since individuals who experienced weight regain or underwent additional interventions did not complete all visits. The absence of a control group following a non‐ketogenic hypocaloric diet does not allow us to determine whether the observed metabolic adaptations and their reversal are specific to VLCKD or reflect a more general response to weight loss and subsequent refeeding. However, the primary objective of this study was not to compare dietary strategies, but to characterize within‐subject longitudinal changes using highly precise metabolic phenotyping. Finally, the assessment of LST was not partitioned into skeletal muscle and organ components with different metabolic rates. This may influence the prediction of energy expenditure and the estimated magnitude of metabolic adaptation. DXA‐derived estimates of LST may be influenced by changes in tissue hydration and composition following significant weight loss. In particular, this may include alterations in the extracellular to intracellular water ratio as those related to glycogen depletion during the ketogenic phase and subsequent repletion during dietary reintroduction, as well as the relative contribution of connective tissue, may affect the accuracy of lean tissue estimation, a limitation that may be further accentuated in the context of ketogenic interventions [44, 45].

In conclusion, women with obesity who completed a 1‐month VLCKD maintained clinically meaningful reductions in body weight and FM over the subsequent 12 months, with only modest losses of LST. Over this period, the initial metabolic adaptation characterized by an early suppression of energy metabolism during VLCKD was progressively reversed and not sustained in the long term. This phenomenon is likely related to the gradual increase in caloric intake during the refeeding phase and to the parallel rise in circulating T3, which may represent a relevant contributing mechanism. The accompanying rise in RER and the shift in substrate oxidation reflect the gradual metabolic transition that occurs as carbohydrates are reintroduced.

The present study provides novel insight into the time course of metabolic adaptation following VLCKD, demonstrating that the early reduction in energy expenditure is not sustained after dietary reintroduction. These findings have important implications for understanding the physiological determinants of weight loss maintenance and support the beneficial role of VLCKD in the control of body weight, when implemented within a structured follow‐up program. However, larger, controlled studies are warranted to confirm these results and to explore their applicability across diverse populations.

## Author Contributions

A.B., P.P., and F.S. designed the study protocol, interpreted the data, and wrote the manuscript. V.A. performed nutritional counseling during follow‐up visits. P.F., C.B., G.P., G.S., L.C., J.K., and A.L. assisted with the interpretation of the data and revised the manuscript. All authors read and critically revised the draft and approved the final manuscript. A.B. and F.S. have full access to all the data in the study and take responsibility for the integrity of the data and the accuracy of the data analysis.

## Funding

This study was sponsored by the University Hospital of Pisa.

## Conflicts of Interest

The authors declare no conflicts of interest.

## Data Availability

Some or all datasets generated during and/or analyzed during the current study are not publicly available but are available from the corresponding author on reasonable request.
